# Food borne illness amongst health care workers, at a Central Hospital, Harare, Zimbabwe, 2016: a retrospective cohort study

**DOI:** 10.1186/s13104-017-3030-x

**Published:** 2017-12-08

**Authors:** Zvanaka Sithole, Tsitsi Juru, Prosper Chonzi, Donewell Bangure, Gerald Shambira, Notion Tafara Gombe, Mufuta Tshimanga

**Affiliations:** 1 Department of Community Medicine, Health Studies Office, University of Zimbabwe, Causeway Harare, P.O. Box CY 1122, Harare, Zimbabwe; 2 Harare City Health Department, Harare City Council, Harare, Zimbabwe; 3 Africa Centers for Disease Control and Prevention, Addis Ababa, Ethiopia

**Keywords:** Food borne illness, Retrospective cohort, Health care workers, Zimbabwe

## Abstract

**Objectives:**

Health care workers (HCW) at a Central Hospital, were served lunch at the hospital canteen on 12 December 2016. On 12 December 2016 at 1700 h, there was a sudden onset of symptoms suggestive of gastrointestinal illness among HCW. We conducted a retrospective cohort study to determine the cause and the factors associated with illness among the HCW at the hospital.

**Results:**

We interviewed 96 respondents. The median incubation period was 6 h (Q_1_ = 4; Q_3_ = 12). Abdominal pain (97.5%) and watery diarrhoea (95%) were the most common symptoms. The majority (97.5%) took antibiotics before collection of stool specimen for analysis, with 24 (60%) of 40 HCW treating themselves. Eating chicken (RR = 44.2, CI 74.07; 95.34) during lunch was associated with the illness. *Staphylococcus aureus* and *Escherichia coli* were isolated from food handlers’ hands, kitchen utensils and work surfaces. *Staphylococcus aureus* was isolated from chicken. None of food handlers had valid medical certificates. One out of four food handlers was formally trained.

## Introduction

Food-borne illness is acute gastroenteritis caused by ingestion of food or drink contaminated with living bacteria or their toxins, or inorganic chemical substances and poisons derived from plants and animals [1]. Common symptoms include abdominal pains, diarrhoea, vomiting, fever, headache and vomiting. The most prevalent pathogens are *campylobacter, salmonella, shigella, hepatitis, brucella, staphylococcus, bacillus cereus, E*. *coli and rotavirus* [1]. Globally, about 1.8 million people died from diarrhoeal diseases, in 2005. Of these deaths, about one-fifth are likely due to food poisoning [2, 3]. Although food-borne illnesses are costly, they are preventable [4].

In Africa, there is limited data to quantify the magnitude of food-borne illnesses. However, the food-borne illness mortality was estimated to be 700,000 persons per year [5]. The high prevalence of diarrhoeal diseases in many developing countries suggests major underlying food safety problems [5]. In Zimbabwe, surveillance systems are in place but incidences of food-borne illnesses are often unreported or undetected. This results in limited data to quantify the magnitude of food-borne illnesses in the country.

On 12 December 2016, between 1230 and 1400, about 96 health care workers (HCW) were served lunch at a canteen. Around 1700 h, among these HCW there was a sudden onset of symptoms suggestive of gastro-intestinal illness. The Harare city response team which included the Public Health Officer, visited the hospital on 13 December, 2016 and did an assessment, which revealed that at least 40 HCW fell ill. We conducted this investigation to determine the risk factors associated with the food-borne illness at the hospital. We hypothesized that consumption of chicken was not associated with the food-borne illness.

## Main text

### Methods

#### Study setting

The outbreak occurred at a central hospital that offers treatment and training services, located in the western district of Harare, the capital city of Zimbabwe. The hospital has 60 student midwives and about 500 student nurses per year. The hospital has three onsite food outlets. The canteen where the outbreak occurred serves lunch and supper.

#### Epidemiological investigation

We conducted a retrospective cohort study. All HCW who ate at the hospital canteen on 12 December 2016 were recruited. We used purposive sampling to come up with the food handlers list. A line list completed by the casualty nurses was used during the investigation. Structured questionnaires were used to ascertain food exposures and occurrence of symptoms. An observational check list was used for an objective assessment for preparedness and response. Epi Info™7 was used to assess the association between the exposure and outcome. Frequencies, means and tables were generated and relative risk (RR) and 95% confidence intervals (CI) were generated.

#### Laboratory investigation

We collected food and water samples. Swabs of food handlers’ hands, kitchen surfaces and utensils, and rectal swabs on food handlers were taken to the government analyst laboratory for analysis of possible pathogens.

#### Environmental investigation

We conducted environmental and sanitary inspections at the canteen to assess cleanliness, waste management, water supply, storage and availability of sanitary facilities. We evaluated the timeliness, quality of preparedness, outbreak detection, investigation and response using a standard checklist adapted from the integrated disease surveillance and response (IDSR) technical guidelines [6].

### Results

#### Demographic characteristics of HCW

Ninety-six health care workers who ate food at the hospital canteen on 12 December, 2016 were interviewed. These include 10 nurses, 17 student midwives, 61 student nurses and eight general hands. Of these, 40 were ill. The median age was 26 (Q_1_ = 25; Q_3_ = 31).

#### Epidemic curve

**Fig. 1:**
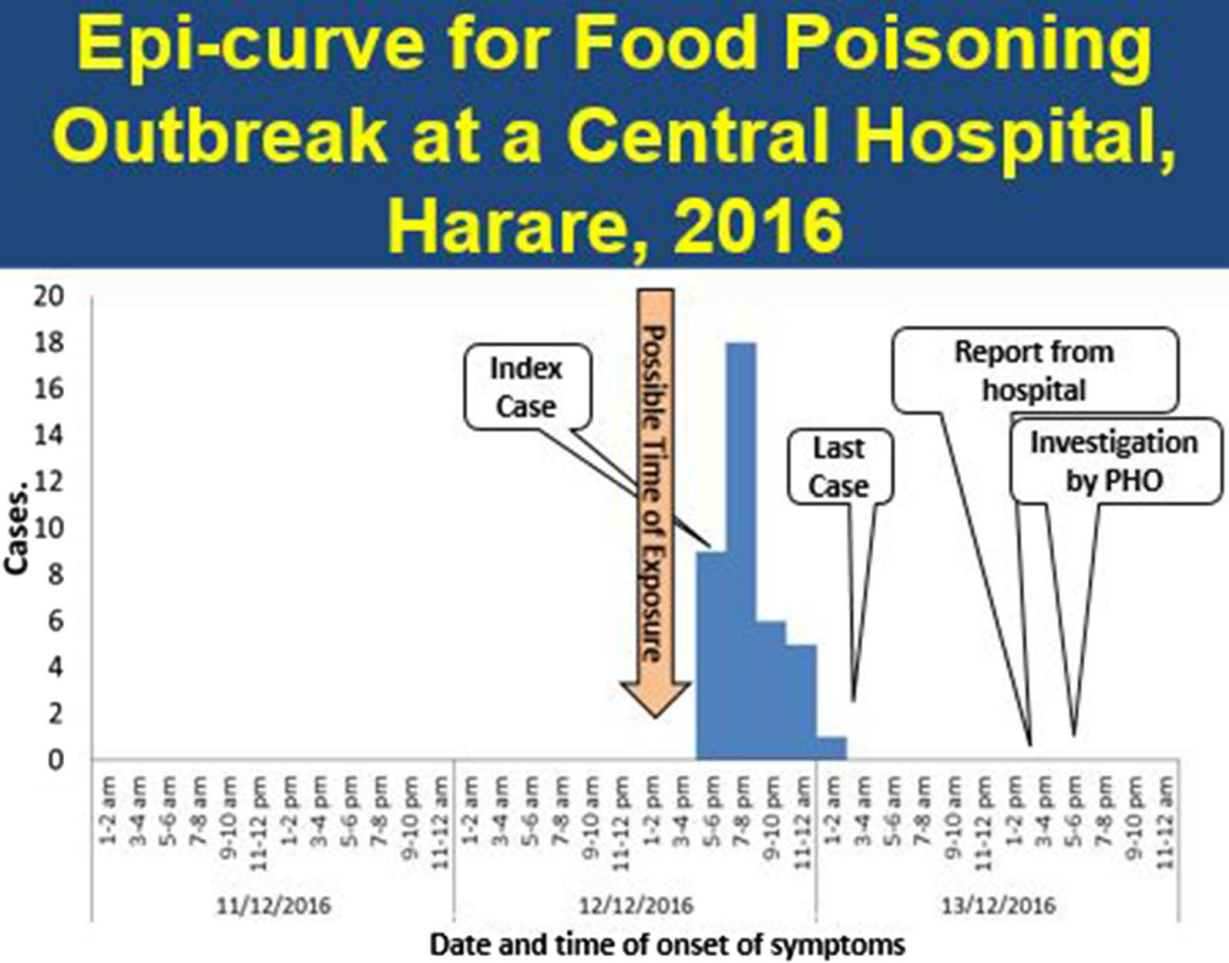
Epi-curve for food poisoning outbreak, Harare, Zimbabwe, 2016

Figure 1 shows the onset of food-borne illness over time. The index case was reported on December 12, 2016 around 1700 h. Thereafter, there was a rise in cases from 7 to 8 pm and a decline from 9 pm to 2 am. The last day of onset on 13 December 2016. The median incubation period was 6 h (Q_1_ = 4; Q_3_ = 12). The outbreak was declared over on 16 December, 2016.

#### Signs and symptoms

**Fig. 2:**
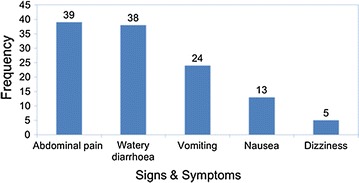
Presenting symptoms of the ill respondents, Harare, Zimbabwe, 2016

Figure 2 summarizes the signs and symptoms presented by the ill HCW. The majority, 39 (97.5%) presented with abdominal pain, and 38 (95%) watery diarrhoea. Vomiting 24 (60%), nausea 13 (13.5%) and dizziness 5 (12.5%) were the least presented symptoms. Twenty-four (60%) of 40 ill respondents treated themselves. Majority of the ill respondents received oral rehydration solution (77%) and ciprofloxacin (62%).

#### Risk factors for contracting food-borne illness

Table 1 presents a bivariate analysis of foods associated with food borne illness among HCWs. The overall attack rate was 42%. Eating chicken (RR = 44.2, CI 6.33; 308.83), soup (RR = 29.07, CI 4.16; 202.99), vegetables (RR = 11.57, CI 4.47; 29.95), and sadza (R.R = 22.5, CI 5.7; 87.8) on 12 December, 2016 was significantly associated with food borne illness. Eating beef (R.R = 0.18, CI 0.03; 1.19), rice (R.R = 0.52, CI 0.19; 1.44), and taking a bottled drink (R.R = 0.13, CI 0.02–0.87) were protective and these were statistically significant. The attack rate of eating chicken (87%) was double the overall attack rate.

**Table 1:** Bivariate analysis of foods associated with food borne illness among health care workers, Harare, December 2016

Exposure variable	Ill		Attack rate	Relative risk (RR)	95% confidence interval	*p* value
	Yes	No				
Sadza						
Yes	38	6	86	22.5	5.7–87.8	0.00
No	2	50	4			
Beef roast						
Yes	1	11	8	0.18	0.03–1.19	0.01
No	39	45	46			
Rice						
Yes	3	10	23	0.52	0.19–1.44	0.15
No	37	46	45			
Chicken roast						
Yes	39	6	87	44.2	6.33–308.83	0.00
No	1	50	17			
Coleslaw salad						
Yes	2	6	25	0.58	0.17–1.97	0.32
No	38	50	43			
Soup						
Yes	39	16	71	29.07	4.16–202.99	0.00
No	1	40	2			
Vegetables						
Yes	36	6	85.5	11.57	4.47–29.95	0.00
No	4	50	7			
Drink						
Yes	1	14	7	0.13	0.02–0.87	0.002
No	39	42	48			
Mineral water						
Yes	2	5	40	0.67	0.20–2.21	0.47
No	38	51	43			

In multivariate analysis, chicken a OR = 73.5 (7.2–752.1) was an independent factor.

The excess risk of illness among respondents who ate chicken was 70 per 100. About 81% of the illness among the respondents who ate chicken can be attributable to eating chicken and could be eliminated if they had not eaten chicken. The excess risk of illness among the total study population was 62 per 100. About 79% of the illness among total study population was attributable to eating chicken during lunch and could be eliminated if chicken was not served for lunch on 12 December 2016.

#### Laboratory results

*E. coli* and *staphylococcus aureus* were isolated from two out of four food handlers hands. *Staphylococcus aureus* was isolated from chicken. A dinner plate, stainless steel work surface, soup ladle, snauffing dish cover, stainless steel sink, chopping board, window seal inside and washing hand basin had numerous to count *E. coli*. *Staphylococcus aureus* was isolated from a dinner plate, stainless steel work surface, wiping cloth, chopping board, rack shelve, window seal inside, stainless steel sink and washing hand basins. There was growth of viable microorganisms in municipal water samples. The on spot water test showed a PH of 7.2 and residual chlorine of 0.1 mg/l.

#### Environmental health audit

##### Food handlers

Four food handlers were involved in food preparation on the 12th of December 2016. One (25%) of four food handlers was formally trained. None had valid medical examination certificates. All food handlers used personal protective equipments. They reported eating the same food as customers and non of them fell ill. The canteen acquires most foodstuffs from reputable licensed food outlets with the exception of vegetables, which come from Mbare market. The catering company was licensed.

##### Kitchen and toilet

Flies were seen in the kitchen. The windows were not fly screened and cleanable from inside. A constant supply of piped hot water, hand washing basins with soap, clean properly maintained sinks and proper water storage facilities were not provided. The deep freezer where meat was stored was small as compared to the volumes of items kept in it. Electricity was reliable though no backup was available in case of electricity cuts. There was one unclean toilet for male and female, staff and patrons. No detergents for toilet cleaning seen. Aprons were stored in the toilet room.

##### Waste management

Refuse and waste were disposed in an open bin. Food leftover meant for dogs was found mixed with fresh items in the same fridge.

##### Emergency preparedness and response of the district

The city had an outbreak emergency kit that could accommodate 300 patients, which was beyond the target of 100 patients. Not all cases reported to health facilities. It took 16 h for Harare Hospital to note the unusual food-borne illnesses and identify the first case. The investigation team took 1 h to respond to the outbreak report.

### Discussion

The epidemic curve shows that all cases occurred within a single incubation period, so the outbreak was caused by a common point source. The common presented signs and symptoms are typical of *Staphylococcus aureus* toxins according to World Health Organization Food-borne Investigation Guidelines of 2008 [7]. The apparent absence of myalgias, chills and fever are more consistent with an intoxication resulting from the presence of toxin in the lower gastro intestinal (GI) tract. Illnesses with short incubation periods, such as enterotoxin producing bacterium, cause upper GI symptoms including nausea and vomiting. The index case was reported just 3 h after the possible exposure and the recovery of all ill respondents within 24 h is consistent with enterotoxin intoxication. This was a point source outbreak, suggesting enterotoxin producing bacterium with a short incubation period and duration of illness, such as *S. aureus,* was the cause.

Even though *E. coli* was isolated from 10 (47.6%) of 21 samples, it is not a probable cause of this outbreak because of its longer incubation period (3 to 8 days, with a median of 3 to 4 days) as compared to the median incubation period in this outbreak. The illness duration of *E. coli* is about 10 days [8], which is longer than the duration of illness in this outbreak. The illness was not waterborne because faecal coliforms were not isolated from municipal water samples.

Contrary to our hypothesis, the chicken served during lunch on 12 December 2016 at the hospital canteen was associated with the food-borne illness. Although there were no other reported outbreaks in Harare west district, the chicken itself may have been a reservoir of bacterium as some studies show *S. aureus* in live chicken [9]. Another source of contamination could have been at the hospital canteen during food handling, preparation or storage. This was a more likely scenario, since *staphylococcus aureus* and *E. coli* were isolated from the food handlers hands, kitchen utensils and stainless steel working surfaces while chicken was contaminated with *staphylococcus aureus*.

At the canteen, sadza with chicken, and sadza with beef cost US$1 per plate. On 12th of December 2016, however, price of sadza with chicken remained at US$1, while price for sadza and beef increased to US$1.50. Therefore most people purchased sadza with chicken.

During the investigation, food leftover could not be found for bacteriological analysis. Howeverr, food samples were taken from the same batch with the chicken consumed by the ill HCWs and *staphylococcus aureus* was isolated.

One possible source of contamination could be that food handlers did not meticulously wash their hands with soap, allowing for cross contamination. *S. aureus* can cause food poisoning when a food handler contaminates the food and then the food is not properly refrigerated [10]. This suggests major underlying food safety problems and indicates the necessity of high quality hygienic conditions during food preparation and storage. Lack of training of food handlers on basic food hygiene may have contributed to this outbreak. Chihava et al. [11], 2012 also described an outbreak of food-borne illness due to staphylococcus intoxication in Bulawayo city restaurant where food handlers were not trained.

Patients treated at Harare hospital who were given loperamide as an anti diarrhoeal drug did not have stool specimens taken for analysis before treatment. This is not in line with national guidelines, which recommend collecting stool specimens before treatment using oral fluids for mild cases during outbreaks [12].

### Conclusion

This was a point source food borne outbreak suggestive of food poisoning due to staphylococcus intoxication. The possible source of contamination was chicken which could be reservoir of bacterium itself or have been contaminated during food handling, preparation or storage. The rapid response team responded promptly to the outbreak. Our findings suggest the need for training of food handlers on basic food hygiene and safety, regular canteen inspections for quality assurance, training of health care providers on case management and ensuring that food handlers are medically examined annually.

## Limitations

No stool specimens were collected. This was because the rapid response team arrived at the hospital an hour after the outbreak report and found all cases having received treatment and were no longer having diarrhoea. No food leftover was found, hence limited certainty about source. However on spot food samples from the same batch with the chicken implicated to the outbreak were taken for analysis. Due to small sample size, our results may not be that precise.

## Abbreviations

HCW: health care workers
CI: confidence interval
RR: relative risk
W.H.O: World Health Organization
C.D.C: Centers for Disease Control & Prevention
